# Appraising Linguistic and Reading Impairments in Kannada-Speaking Persons With Central Alexia

**DOI:** 10.7759/cureus.63437

**Published:** 2024-06-29

**Authors:** Akshaya Swamy, Sathyapal Puri Goswami

**Affiliations:** 1 Department of Speech and Hearing, Father Muller College, Mangalore, IND; 2 Deaprtment of Speech Language Pathology, All India Institute of Speech and Hearing, Mysuru, IND

**Keywords:** correlation, reading comprehension, oral reading, alexia, aphasia

## Abstract

Introduction: Alexia is an acquired condition resulting in impaired abilities to comprehend and/or read aloud written scripts secondary to lesions in the brain involved in reading processes. Just as how linguistic aspects are multi-faceted in persons with aphasia (PWAs), the reading impairments also vary extensively across each PWA depending on the type and nature of the language deficits. Each language has its unique linguistic properties. Whether the impairment is in the spoken form or written form, the dissociations in both aspects across the linguistic distinctions are paramount. Given the diverse orthographic features of Kannada, alongside inquiries into the impairment in oral language skills among individuals with acquired reading difficulties, the study emphasizes distinguishing the concurrent language processes associated with reading impairment.

Aim: The study aimed to explore the relationship between spoken language and reading abilities in Kannada-speaking post-stroke survivors with acquired alexia.

Method: The study recruited 15 Kannada-speaking adults with complaints of reading impairment post-ictus through convenient sampling. The enrolled participants included both males and females with a mean age of 42.2 years (SD=15.58; range=20-68) with 16 years of formal education. The study performed three primary investigations; a) linguistic, b) oral reading, and c) reading comprehension. Linguistic tasks entailed semantics, syntax, and phonological tasks. Oral reading entailed real word and non-word reading tasks. Reading comprehension tasks entailed single-word level, sentence-level, and paragraph-level tasks.

Results: Results of overall domain performance across linguistics, oral reading, and reading comprehension revealed superior performance in linguistics (M=71.77, SD=19.18) followed by reading comprehension (M=70.55, SD=24.10) and oral reading (M=41.55, SD=24.66), which was significant (p<0.05). Performance in phonology was weakest (M=58.06, SD=12.44) compared to syntax (M=71.13) and semantics (M=85.33) on comparing PWAs’ abilities within the linguistic domain. Reading abilities were compared across oral reading and reading comprehension, which significantly varied (p<0.05), and reading comprehension abilities were superior (M=72.65, SD=24.10). Task-specific variabilities were significant (p<0.05) in both oral reading and reading comprehension, wherein, real word reading (M=58.22) and comprehension at a single level (M=80.22) were better performed. Results of correlation analysis revealed semantics (r=0.494, p<0.05) and phonology (r=0.428, p>0.05) were highly positively correlated to oral reading abilities. Syntax (r=0.412, p>0.05), and semantics (r=0.377, p>0.05) were strongly positively correlated to reading comprehension abilities.

Conclusion: The study has convincingly shown that performance on reading-related tasks reflects the functionality of central semantic, phonological, and syntactic processing elements. The literate adults routinely interact with both spoken and written language and a comprehensive assessment framework of language processing must encompass both modalities (linguistic and reading) for individuals with alexia, specific to Indian languages. Owing to the transparent writing system of the Kannada language, a multimodal phonological and lexical-based reading treatment may prove to be beneficial in remediating reading impairments among adult post-stroke survivors.

## Introduction

Acquired reading impairment or alexia has been studied with major emphasis on identifying the neurological bases or cognitive processes (like visual processing, attention, working memory, etc) in the recent past. Wherein, the neuro-anatomical approaches aim to explicate the corresponding neuroanatomical bases specific to the type of reading impairments. The neurological bases for oral language and reading are exclusive to each other. In language tasks, Wernicke’s area is observed to present peaks of activation when connected with Broca’s area via the arcuate fasciculus. For written language, the visual region of the fusiform gyrus (BA 37) may constitute orthographic representations of words, and the superior parietal lobe (BA 7) could play a role in spatial attention aspects of reading. Wernicke’s area (BA 22) and its adjacent areas, including the angular gyrus and supramarginal gyrus (BA 39, 40), are multimodal areas possibly responsible for integrating spoken and written word forms, along with their associated meanings or semantics [1]. Additionally, the variabilities in the neural networks across tasks may be presumed as a consequence of contrasts in the degree to which semantic, phonologic, syntactic, or orthographic processes are retrieved, which may be mirrored due to minute distinctions in the brain activation [2]. Karanth opined that the cognitive models strive to rationalize the effect of cognitive processes like visual perception, and/or other cognitive processes hampering the reading processes [3]. Parallel to these, there are extensive attempts to explain these processing deficits in reading through behavioral or neuro-computational models. Wherein, these models attempt to ascertain the relationship between a set of processing stages of cognition involved in reading and the neuronal network of pathways.

Justifying the deficits of individuals with aphasia and acquired alexia is quite overwhelming through neuro-computational models, owing to the heterogeneous nature of the condition. As commonly observed in many language models, the gap between aphasia and alexia research can be attributed to the traditional notion that written and spoken language are supported by separate neural processes [4]. Spoken language models [5] typically overlook the significance of orthographic abilities, while written language or reading models [6] fail to address the influence of spoken language skills on reading. Although some models [7] incorporate both spoken and written language processing, they still depict spoken and written language as relying on distinct components specific to each language modality [8].

In contrast to the notion of separate systems for spoken and written language, a parallel-distributed processing (PDP) connectionist model for single-word processing [9-11] proposes that processing spoken and written words involves the simultaneous activation of semantic, phonological, and orthographic units [8]. According to the PDP model, lexical processing results from the concurrent processing of semantic, phonological, and orthographic information, which interact simultaneously to form a learned pattern of activation. The connectionist/triangle model is structured around three pathways: semantics-phonology, phonology-orthography, and orthography-semantics, all known to have a significant contribution to language processing. As language processing evolves and becomes more efficient, a division of labor emerges. A much more specific and conclusive interaction between language and reading processes was proposed through the primary system hypothesis (PSH).

According to the connectionist functioning of language processes, the PSH ascertained that the connected primary neural systems (visual, semantic, and phonological interact collectively in the process of reading all types of words (regular, irregular, and non-words). The model further hypothesized that disruption in reading results from a deficit in one or more of these modality-independent processes [4,8,10,11].

Concerning the PSH, the authors elaborated on the relationships between impaired oral reading abilities and semantic and phonological impairment. Normal reading abilities are attributed to intact semantic and phonological processing. Phonological alexia is a result of poor non-word reading with relatively better regular word reading skills, which is attributed to a higher degree of impairment in phonological processing according to PSH. Deep alexia, is attributed to a severe form of phonological impairment, resulting in severe deficits in phonological processes and mild semantic impairment as per PSH [12]. Crisp and Lambon Ralph termed global alexia, associated with severe semantic and phonological deficits, characterized by absolutely diminished reading abilities (not even letter recognition) [13].

Thus, the PSH presumes that later-acquired reading abilities evolve from and depend on the same cognitive mechanisms akin to earlier-acquired spoken language abilities. Henceforth, significant relationships between spoken and written language impairments are predictable [8,12]. However, a major source of works on acquired reading impairments sprout from English-speaking individuals, and these attempts mainly focus on explaining the peculiar nature of the English language, which follows an alphabetic system of orthography. In specific interests of the study, Dravidian languages are embedded with alpha-syllabic orthography, which is a much more transparent system as opposed to English orthography. For example, in the English words "king" and "kite," /ki/ represents two different phonemic correspondences in these words. However, in Kannada or in any Dravidian language, /ki/ and /kai/ have distinct transparent phoneme-grapheme correspondence. Also, /s/ had dual grapheme representation in /truce/ and /sauce/. This is not so in Dravidian languages, there is a single transparent grapheme representation exclusive to each phoneme (or sound).

In this context, Coltheart et al. suggest that surface dyslexia cannot exist in languages with regular/transparent orthographies, referring to the Italian language [6]. In the Indian context, extensive efforts and studies are conducted on the Indian population by Karanth [14-16], who proved the statement of Coltheart et al. suggesting that surface dyslexia cannot exist in the case of Dravidian languages, owning to transparent orthography in the majority of them [6]. In specific, Kannada is a Dravidian language, written in a phonetically regular script. The script has a 50-letter alphabet called "akshara" and involves a large number of regular and irregular rules while forming syllables. Linguistically, syllables can be dissected into onset, nucleus (vowel), and coda components. In Indian orthography, a letter unit (or "akshara") may denote a standalone vowel (V), a consonant-vowel syllable (CV), or consonant clusters with a vowel, such as CCV or CCCV. The coda portion of a syllable in a word consistently pairs with the subsequent letter [17]. For example, the syllabic breakdown of the word "prarthane" ("prayer") could be /prar/, /tha/ and /ne/. This word comprises three letters or "aksharas," /prar/, /tha/ and /ne/.

The primary set of letters, known as "aksharamala", representing a sequence of letters, includes vowels and consonants in their fundamental forms. The secondary forms of vowels are employed in diverse CV sequences (excluding C+/a/ combinations). Concerning the transparency-opaqueness continuum, Indian writing systems lean towards transparency, with letter sounds and names displaying nearly perfect correspondence [11].

Karanth reported cases of pure alexia with differential impairment in Kannada and English languages [14]. The differential nature of alexia in these languages was attributed to distinct language systems, the pattern of language acquisition, and the separate neural bases involved in processing different scripts [12-14]. Similarly, Ratnavalli et al. opined that the dissociations and degree of impairment differ across Dravidian languages and English [17]. They suggested future research must establish explanations based on the reading models and processing exclusively for Dravidian languages [17]. Given the diverse and intricate orthographic features of the Kannada language, alongside inquiries into the extent of impairment in oral language skills among individuals with acquired reading difficulties, the study emphasizes the importance of distinguishing the concurrent language processes associated with reading impairment, thereby supporting the PSH. Such investigations are essential for explaining the processes inherent in reading Indian language orthographies.

Aim: The study aimed to explore the relationship between spoken language and reading abilities in Kannada-speaking post-stroke survivors with acquired alexia. Specifically, the study was curious to explore a) the effect of alexia on performance across components of linguistics (semantics, phonology, and syntax), b) the effect of alexia on the performance of reading (oral reading and reading comprehension), and c) the influence of semantics, syntax, and phonology on oral reading and reading comprehension abilities in acquired alexia.

## Materials and methods

Participants

The study recruited 15 Kannada-speaking adults with complaints of reading impairment post-ictus through convenient sampling. The enrolled participants were males and females with a mean age of 40.33 years (SD=15.53; range=20-68) and all possessed formal education of 15.77 years on average (S.D=1.79; range=12-18). All the participants were assessed with the Kannada version of the Western Aphasia Battery (WAB-K) to confirm the diagnosis of aphasia (Table 1).

**Table 1:** Demographic details of all participants with aphasia enrolled in the study MPO: months post-stroke onset

Patient	Age	Gender	Education (in years)	Qualification	MPO (in months)	Aphasia quotient
P1	22	Male	16	Graduation	26	82.5
P2	46	Male	15	Graduation	45	67.7
P3	20	Female	16	Discontinued graduation	12	59.1
P4	32	Male	18	Post-graduation	10	80.5
P5	34	Male	18	Post-graduation	17	75.8
P6	56	Female	18	Post-graduation	34	86.2
P7	24	Male	14	Discontinued graduation	16	70.6
P8	48	Male	12	Higher secondary	9	45.6
P9	37	Male	15	Discontinued graduation	28	66.3
P10	57	Male	16	Graduation	18	66
P11	59	Female	16	Graduation	40	40
P12	48	Male	18	Post-graduation	23	56.5
P13	68	Male	16	Post-graduation	17	87.4
P14	34	Male	14	Graduation	24	45
P15	20	Female	14	Graduation	6	78

The study abided by strict inclusion and exclusion criteria. PWAs with left cerebrovascular accident (CVA), with adequate auditory verbal comprehension abilities (greater than 4 on WAB-K), and who possessed right-handedness pre-morbidly were recruited in the study. Individuals with a history of developmental dyslexia or any other neurological ailments other than left hemisphere ictus and/or individuals with visual impairment or visual neglect were excluded from the study. All participants were administered with language proficiency scale to note their proficiency level in their native language- Kannada. Additionally, through this, we noted the bi/multilingual exposure, if present. In this instance, individuals with language proficiency less than 30-50 in the second and third language, and proficiency above 60 in Kannada (native language) were only recruited in the study using the Language Experience and Proficiency Questionnaire (LEAP-Q) [18]. All participants gave informed consent to take part in a multisession language assessment using protocols approved by the Ethics Committee for Bio-Behavioral Research Projects Involving Human Subjects at the All India Institute of Speech and Hearing, University of Mysore, Mysuru, India (Approval No.: DOR.9.1/PhD/AS/926/2021-22; dated 08.12.2022).

Procedure

All assessments were carried out in a quiet room, and free from distractions. The study protocol entailed three primary investigations: a) linguistic tasks, including semantics, phonology, and syntax in Kannada; b) oral reading tasks at single word level, including real word reading, and non-word reading in Kannada; and c) oral reading comprehension across single word level, sentence level, and paragraph level in Kannada.

Tasks-specific instructions were conveyed to every PWA before the administration of each task (Appendix A). Also, trial stimuli were presented for every task to familiarize them with the task. The written stimuli (black and bold, 42 font size), and colored pictures were presented over the desk in printed format on an A4 size flash card. While carrying out the tasks no specific cues were rendered. No time limit was imposed while testing, and authors refrained from response-contingent feedback. The tasks were counterbalanced wherein, one-half of the PWAs were subjected to linguistic tasks first and the other half to reading tasks next, to nullify the effect of stimuli complexity and performance load on the PWAs.

Each domain, namely semantics, syntax, and phonology of spoken language entailed sub-tasks. The tasks were least loaded with verbal responses, in order to reduce the speech motoric response load on the individual. Likewise, the reading domain entailed sub-tasks of oral reading and reading comprehension. These tasks and sub-tasks are depicted in Table 2. 

**Table 2:** List of tasks and sub-tasks compiled in linguistic and reading domains for the study protocol

Domains	Semantics	Phonology	Syntax
Linguistic	Picture association	Minimal pair judgment	Comprehension of plural forms
	Picture matching	Real word rhyme judgment	Comprehension of tense markers
	Auditory comprehension	Non-word judgment	Spoken sentence to picture matching
	Auditory judgement	Parsing/blending sounds	Sentence completion with locatives
Reading	Oral Reading	Reading Comprehension	
	Real word	Word level, sentence level, paragraph level	
	Irregular word		
	Non-word		

All the tasks, sub-tasks, and stimuli were compiled from various tasks of standardized test batteries. The Kannada stimuli set was subjected to well-experienced (above five years) Kannada-speaking speech-language pathologists (SLPs) for appropriateness, stimulability, imageability, frequency, and sensitivity. Stimuli were rated based on a 3-point rating scale, where "zero" signified "least relevant" and "two" signified highly relevant on attributes like "simplicity," "size of the picture," "image clarity," and "image appropriateness" for pictures. For printed stimuli, they were judged on "lexicality," "syllable length," and "appropriateness" of the words. Final stimuli were compiled using point to point comparison method and the set that received scores above 80% on inter-judge and intra-judge ratings were considered for the final stimuli.

Scoring

Each item on each task received a raw accuracy score (1=correct; 2=incorrect). Self-corrections were permitted and the participant's final response was considered. The total raw score of each task was converted into percentile score (e.g., picture association percentile score = average of total raw score/total max score of the task x 100). This standard scoring was ensured as the total items varied across sub-tasks, and also to nullify the effect of stimuli complexity to perform across the tasks. The percentile scoring was considered for descriptive and specific tests of statistical hypothesis. For correction analysis, the raw scores of the data were analyzed.

The study investigated two main domains, linguistics and reading. Thus, the overall linguistic and reading scores of all 15 participants were computed. Subsequently, average percentile scores for specific linguistic subdomains, namely; semantics, syntax, and phonology were computed. Similarly, in the reading domain, average percentile scores for oral reading and reading comprehension were calculated. These scores were further subjected to specific and detailed statistical analysis.

We also explored the reading abilities of individuals to understand the alexia profiles. This involved calculating the deviances in accuracy between real words and non-words, thereby obtaining a lexicality measure for each individual's reading proficiency. To ascertain the differences across reading comprehension, single-word versus sentence versus paragraph reading comprehension was also compared and contrasted.

Statistical analysis

The obtained data (average percentage scores) of domains of linguistics and reading and sub-domains were initially analyzed for descriptive statistics. Mean (M) and standard deviation (SD) were computed for measures of linguistics (semantics, phonology, and syntax) and reading (oral reading and reading comprehension) in Kannada-based tasks. The data was then subjected to Shapiro-Wilk's test for normality, and the data significantly followed a normal distribution (p>0.05). Therefore, parametric repeated measures ANOVA was employed to see the main effect of domains (linguistics versus reading); and the main effects of sub-tasks of reading (oral reading versus reading comprehension) in Kannada on the percentage scores. when there was a significant influence (p<0.05) of tasks on scores observed, the least significant difference (LSD) method was applied to analyze the pairwise significance between the variables.

Further, the one-way repeated measure ANOVA was applied to the linguistic domain to observe the main effect of the subdomains (semantic, phonology, and syntax) in the Kannada language. When there was a significant influence (p<0.05) on the variable observed, the LSD method was applied to analyze the pairwise significance between the variables. Similarly, the reading domain was subjected to a paired sample t-test to analyze differences between reading subdomains (oral reading versus reading comprehension). Task-specific differences were further addressed through the one-way repeated measure ANOVA with post hoc analysis for reading comprehension tasks (single word versus sentence versus paragraph reading). A paired sample t-test was applied to oral reading tasks (real word versus nonword).

Further, to ascertain the relationship between language and reading tasks, Karl Pearson's correlation analysis was employed. The statistical significance value was compared with 0.05 or 0.01 level of significance. The entire statistical analysis was carried out using IBM SPSS Statistics for Windows, Version 23.0 (IBM Corp., Armonk, NY).

## Results

The study investigated three main objectives; a) to explore the variabilities in performance across components of linguistics (semantics, phonology, and syntax), b) to explore the components of reading abilities (oral reading and reading comprehension), and c) to explore the correlation between linguistic and reading comprehension abilities. 

Overall domain performance across linguistics, oral reading, and reading comprehension

The effect of overall domain performance across linguistics, oral reading, and reading comprehension was analyzed through the one-way repeated measure ANOVA. The results suggested a significant effect (p<0.01) of performance on various domains (F {2,28}=12.21, p=0.000). Pronounced performance was noted in both linguistics and reading comprehension compared to oral reading abilities on observing the overall mean scores as delineated in Table 3.

**Table 3:** Overall performance in linguistics, oral reading, and reading comprehension

Domains	Mean	SD
Linguistic domain	71.77	19.18
Oral reading	41.55	24.68
Reading comprehension	72.65	24.10

Performance in specific domains of linguistics; semantics, phonology, and syntax

**Figure 1:**
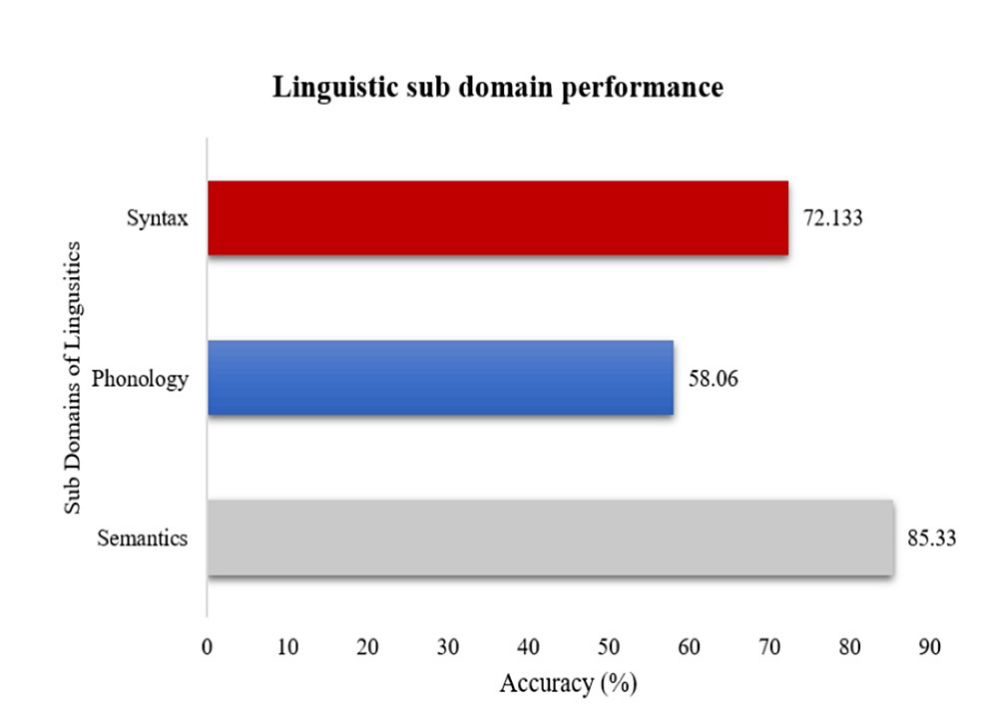
Mean scores of linguistic subdomains

The effect of subdomain performance of linguistics across semantics, phonology, and syntax were addressed through the one-way repeated measure ANOVA. The results revealed a significant effect of performance (p< 0.05) on subdomains of linguistics (F {2,28}= 23.00, p=0.00), suggesting that there is variability in performance across semantics, syntax, and phonology. Subsequently, the post hoc pairwise analysis was applied through the least significant difference (LSD) method, and results showed a significant difference between semantics versus syntax versus phonology as shown in Table 4. As illustrated in Figure 1, PWAs outperformed in semantics, followed by syntax, and performed poorer in phonology in linguistic domains of Kannada. 

**Table 4:** Linguistic subdomains; pairwise comparison * Indicates significant at p< 0.05, ** Indicates significant at p<0.01

Pairs	Mean difference	p-value
Semantic vs phonology	27.067^**^	0.00^**^
Semantic vs syntax	13.000^**^	0.00^**^
Phonetics vs syntax	-14.067^*^	0.05^*^

Performance in overall reading; oral reading and reading comprehension

As mentioned earlier performance of the overall domain across linguistics, oral reading, and reading comprehension was analyzed through one-way repeated measure ANOVA. The results suggested a significant effect (p<0.01) of performance on various domains (F {2,28}=12.21, p=0.000). Further, post hoc analysis was applied through the LSD method, and results showed a significant difference (p< 0.01) across oral reading versus reading comprehension (mean difference=-31.101, p=0.001). Wherein, it was noticed that reading comprehension (M=72.65, SD=6.25) was better than oral reading (M=41.55, SD=6.3) on comparing the mean scores.

Performance in specific tasks of oral reading and reading comprehension

**Figure 2:**
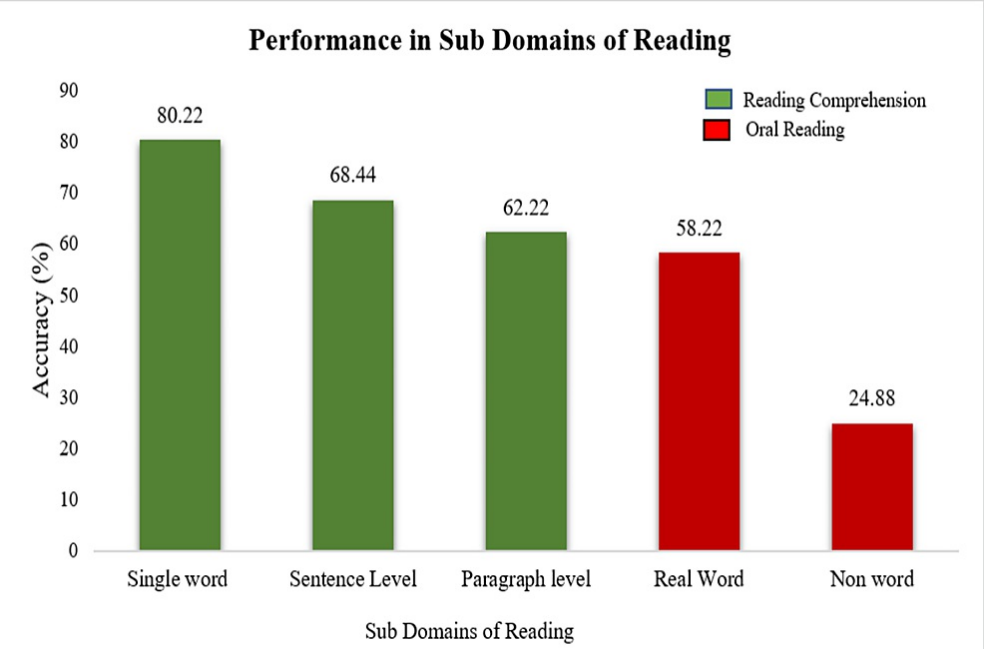
Mean scores of reading subdomains

To evince the lexicality effect on oral reading abilities, the scores of real word reading and non-word reading were compared through paired sample t-tests. Results revealed real word reading significantly differed with non-word reading abilities (t {14}=5.61, p=0.00). Specifically, on observing the means scores, performance in the real word reading was superior to non-word reading (Figure 2). 

Similarly, the difference in performance across tasks of reading comprehension was analyzed through the one-way repeated measure ANOVA, and results revealed significant differences across single word, sentence level, and paragraph level (F {2,28}=11.28, p=0.000). The post hoc analysis is shown in Table 5. Specifically, on observing the descriptive measure, the performance in single-word reading was exponentially better than in sentence and paragraph. Paragraph reading was noted to have the poorest scores (Figure 2).

**Table 5:** Reading comprehension; task-specific, pairwise comparison * Indicates significant at p<0.05

Pairs	Mean difference	p-value
Single word vs sentence	11.55^*^	0.012^*^
Single word vs paragraph	17.78^*^	0.002^*^
Sentence vs paragraph	6.22^*^	0.008^*^

Correlation between linguistic subdomains and reading subdomains

The study explored the relationship between linguistic subdomains and reading subdomains on the raw scores of the participant's performance in the study. Karl Pearson's test of correlation revealed a positive correlation between overall linguistic performance and overall reading, which was significant (r=0.652, p=0.008).

**Figure 3:**
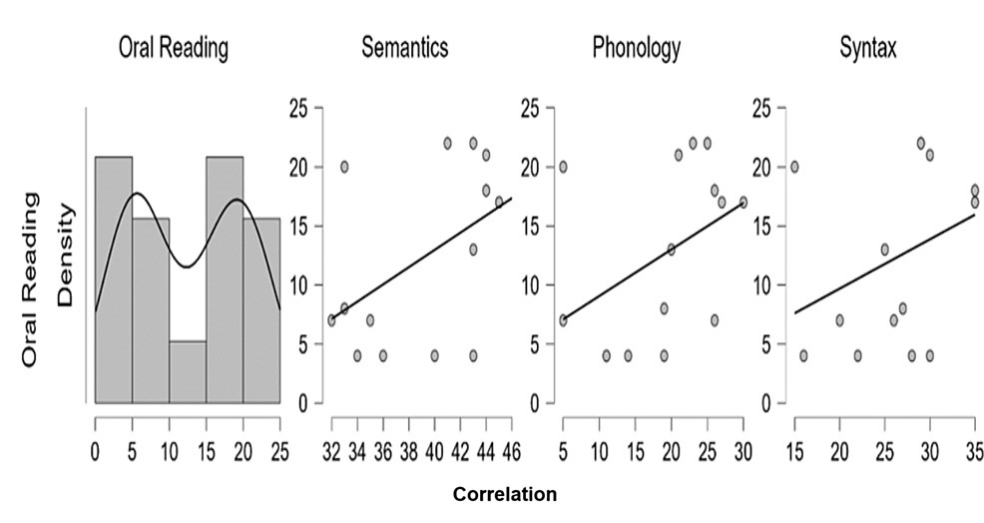
Relationship between oral reading and semantics, phonology, and syntax

Specifically, each linguistic subdomain, that is, semantic, phonology, and syntax was analyzed for correlation with oral reading. It is remarkable to note the positive correlation between semantics, syntax, and phonology with oral reading in Kannada; wherein, semantics was highly correlated with oral reading (r=0.494, p>0.05), followed by phonology (r=0.428, p>0.05) and syntax (r=0.361, p>0.05). Therefore, the trend suggests that oral reading is directly compared to semantic>phonology>syntax in Kannada (Figure 3).

**Figure 4:**
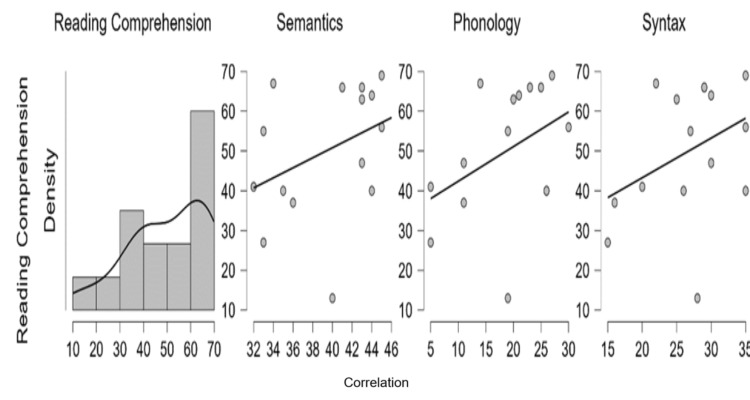
Relationship between reading comprehension and semantics, phonology, and syntax

Correspondingly, reading comprehension abilities were correlated with linguistic subdomains. Reading comprehension in Kannada was predominantly positively correlated with syntax (r=0.412, p>0.05), followed by semantics (r=0.377, p>0.05) and phonology (r=0.372, p>0.05) which was marginally less to semantics (Figure 4).

## Discussion

Effect of alexia on performance across linguistics, oral reading, and reading comprehension

The study initially investigated the overall performance differences in the linguistic domain, oral reading, and reading comprehension abilities through the mean scores of all the 15 PWAs. The results revealed superior performance in linguistics (M=71.77, SD=19.18) and reading comprehension (M=72.65, SD=24.10) compared to oral reading (M=41.55, SD=24.68). Also, the main effects revealed significant differences across these domains.

The superior performance in linguistic abilities compared to oral reading abilities may be attributed to the exclusive neurological bases for oral language and reading. For reading the neurological bases are much wide spread and complex. Thus, recovery from these neurological bases may be difficult and time-consuming. The dominance in performance on linguistic tasks in the study over the reading tasks may be attributed to extensive usage and exposure to spoken language mode. Also, the performance variability may be attributed to the higher cognitive load bound in oral reading tasks compared to linguistic tasks.

The superior performance in linguistic tasks observed in the study compared to reading tasks may be attributed to the extensive usage and exposure to spoken language in everyday life. The majority of individuals primarily use spoken language mode for their daily communication needs. Reading or literacy skills are infrequently utilized in daily activities unless one is engaged in desk work, academics, or keeps up with current events through newspapers or social media. Additionally, individual preferences for reading and writing vary based on social factors such as interests, literacy level pre-stroke, socioeconomic status, family background, and occupation [19]. Variations among individuals may also arise from inherent differences in the importance placed on reading and individual reading preferences [20]. Spoken language or linguistic processing tends to remain well preserved or recover more effectively after stroke compared to reading abilities.

Alleviated performance in linguistic tasks compared to reading tasks among the participants could be explained by variations in task complexity. In the research, some PWAs demonstrated proficiency in basic linguistic evaluations but encountered difficulties with written language tasks (reading tasks). For these individuals, deficits in the written language mode may arise from increased cognitive demands linked to written text comprehension [21]. Moreover, deficiencies in working memory, attention allocation, and executive function, essential for processing written content, might impact their overall reading performance. The observations align well with the findings by Chesneau and Ska [22].

The study witnessed that even the reading comprehension abilities were equivalent to performance in overall linguistic tasks, better than oral reading abilities. Concerning the nature of the task, oral reading requires active central phonological processing, visual perceptual skills, and peripheral sensorimotor processing for oral output. In contrast, reading comprehension doesn't demand oral output. Various interactive processes such as visual perception, decoding graphemes, working memory, attention, and metacognitive abilities are involved in comprehending written text [20]. However, the cognitive load is comparatively lower compared to oral reading output. Specifically, the study included tasks such as single-word comprehension, sentence comprehension, and paragraph comprehension, which involved activities like written word-picture matching, sentence-picture matching, and paragraph-to-picture matching, respectively. Thus, owing to the degree of cognitive load involved in performance in various reading tasks, oral reading tends to be more taxing compared to reading comprehension. Among reading comprehension tasks, comprehension at the single-word level demands the least cognitive load compared to sentence and paragraph levels.

Effect of acquired alexia on performance across sub-tasks of oral reading and reading comprehension

The study attempted to understand the task-specific dissociations in the performance of all PWAs on oral reading and reading comprehension abilities. Particularly, the study discerned significant elevated performance in real word reading tasks compared to non-word reading within the oral reading tasks, suggesting PWAs with Alexia have well-preserved or better recovered oral reading abilities in real word context in contrast to non-word oral reading skills. This finding is supported by the findings of Ratnavalli et al. [17]. They compared alexia and aphasia in Kannada-English bilinguals and observed that patients were making distinct types of reading errors in each language. In Kannada, the errors predominantly involved non-words, sparing real word reading. Thus, examining oral reading abilities through non-words may be a most sensitive task to understand the severity of reading deficits in the Kannada language. 

As per Raspcsak et al., a notable characteristic of written language syndromes is identified as the lexicality effect, wherein real words are read or spelled with greater accuracy compared to non-words. This occurs because non-words necessitate the conversion of letters to sounds and vice versa. They illustrated that the fundamental deficit lies in a general phonological impairment, not confined solely to reading or spelling [23]. This impairment manifests in tasks demanding phonological awareness and manipulation skills, in addition to orthographic knowledge. This is well reflected in the performance in linguistic abilities, wherein phonology was drastically affected among the PWA cohort of the study. Consequently, their oral reading abilities in non-words are much compromised, suggesting an inherent connection between linguistic and reading abilities.

In the Kannada language that encompasses transparent orthography, for reading real words, the lexical route is involved with the contribution of both phonology and semantics. Non-words, being unfamiliar words encountered for the first time, lack stored meanings in the internal orthographic lexicon. Consequently, they necessitate processing through the phonological pathway, involving the conversion of phonological stimuli to orthography on a one-to-one basis [24]. However, if there is damage to the phonological route responsible for converting phonemes into graphemes, reading and writing non-words may become challenging. Thereby, based on the Dual Route Cascaded model, poor non-word reading abilities reflect the incompetence of the phonological route. While semantics is well preserved, the real word abilities tend to be read comparatively better owing to the above reason through the lexical route. Thus, this proves that there is a lexicality effect in reading Kannada orthography.

Yet another explanation attributed to compromised non-word abilities could be due to the cognitive load it encompasses. As per the aforementioned discussion, reading real words involves one-to-one grapheme-phoneme correspondence, especially in a transparent language like Kannada in both reading and writing. Consequently, the formation of lexical-semantic representations entails less burden, albeit phonological adjustments are necessary. However, with non-words, the processing load escalates, potentially necessitating additional cognitive processes such as suppression and attention compared to regular words while judging the non-words [25]. It is also acknowledged that individuals with aphasia experience heightened processing demands due to damage to the language center and impaired cognitive function [26].

Further, the study explored the difference between reading comprehension abilities in the Kannada language. Results manifested clearly significant dominance in performance in single word comprehension (M=80.22, SD=5.78) compared to sentence level (M=68.44, SD=7.04) and paragraph level (M=62.22, SD=7.41). This finding is in line with Caplan and Evans, who opined that difficulty in sentence comprehension will invariably affect paragraph comprehension [27].

These outcomes were likely shaped by the interaction of two primary components in cognitive-linguistic processing: the impact of the cognitive systems facilitating lexical processing and how these systems were taxed by the varied tasks. Certainly, cognitive functioning among PWAs is compromised and, thus affects reading comprehension abilities across single word, sentence level, and paragraph level. Consequently, paragraph level tends to be more cognitively taxing and necessitates higher cognitive functioning, and thus their performance is compromised compared to least taxing single-word reading comprehension. However, orthographic-specific studies are warranted to ascertain the variabilities across levels of tasks on reading comprehension abilities.

Effect of linguistic components on oral reading and reading comprehension in PWAs with Alexia

The study unraveled the relationship between semantics, phonology syntax, and oral reading and reading comprehension based on the raw scores of the participants' performance. The test of correlation revealed a positive correlation among semantics, phonology, and syntax with oral reading and reading comprehension. This finding was consistent with a study by Webster et al., who also found a strong positive correlation between language impairment and reading impairment through the administration of the Porch Index of Communicative Ability [21].

In particular, the correlation between semantics and phonology with the oral reading task was notably strong (r=494, p<0.05 and r=428, p>0.05, respectively), indicating their role as reliable predictors of oral reading proficiency. This suggests that any impairment in either semantics or phonology could detrimentally affect oral reading abilities, and vice versa. This observation finds substantial support in various studies employing priming paradigms. Notably, poor readers exhibited larger semantic priming effects compared to proficient readers in contexts where target words followed a single word or sequential priming. Moreover, Booth et al. demonstrated a significant positive correlation between speech production, semantics, phonological skills, and oral reading performance, with orthographic and phonologic priming showing notable associations with naming accuracy [2]. Adding to this, a recent study by Beeson et al. reinforced the notion that semantics, phonological ability, and speech production are robust predictors of both spoken naming and oral reading performance [28].

Several authors have proposed neurocomputational models of language processing that illustrate dissociations in linguistic aspects and their impact on reading. Plaut and Booth observed inherent individual differences in semantic processing among poor readers, which impeded grapheme-phoneme connections [29]. Consequently, these semantic connections influenced a decrease in the speed of word recognition processes. Prominent computational models in current reading-aloud literature presuppose that interactive activation serves as the predominant mode of processing dynamics, particularly within the lexical system [6,10-11].

In the context of the oral reading task, there is a notable sharing of inherent neural pathways between semantics and phonology. This observation is supported by comparisons of reading profiles in phonological alexia and surface alexia [23-25]. Researchers suggest that the reading deficits observed in these conditions reflect variations in the central phonological and semantic systems of language, which depend on neural networks. The existence of a dorsal pathway is firmly established to support phonology, speech production, phonological short-term memory, and awareness. Furthermore, the semantic network is widely distributed, involving contributions from regions such as the left anterior inferior frontal gyrus (pars orbitalis) and angular gyrus within the left middle cerebral artery distribution, along with certain regions in the right hemisphere. Thus, semantics and phonological processing play crucial and distinct roles in the oral reading process.

In the specific interest of the study, according to the PSH [11] and the parallel distributed processing (PDP) [30] model of reading, both typical and atypical reading patterns are attributed to a strong interaction between the semantics and phonological systems [13]. Reading impairments would thus result from deficits in these systems, which may manifest not only in phonological and semantic pathways but also depending on the status of general cognitive systems, which are not exclusive to reading [13]. This study also explores the influence of non-reading abilities, such as semantics, phonology, and syntax (linguistic abilities) on reading abilities, highlighting the inherent interaction between linguistic modalities in reading proficiency.

Furthermore, the study attempted to explore the effect of semantics, syntax, and phonology on reading comprehension. The results indicated a strong correlation between reading comprehension and syntax (r=0.412, p<0.05), followed closely by semantics (r=0.377, p>0.05), with phonology slightly less correlated (r=0.372, p>0.05) compared to semantics. These findings support the notion that shared syntactic, phonological, and semantic processing mechanisms contribute significantly to the comprehension of both spoken and written sentences [8]. Additionally, given that reading comprehension tasks involve processing at word, sentence, and paragraph levels, syntactic knowledge and processing are inherently necessary to comprehend text at the sentence level. Following syntactic processing, which involves syntax verification and judgment, semantic processing becomes crucial for determining the appropriateness of the text's meaning to achieve complete sentence comprehension. In short, syntax could be a much more sensitive and strong linguistic predictor of reading comprehension followed by semantics and phonology. 

In a nutshell, based on the PSH and the PDP model of reading, both typical and atypical reading patterns in the Kannada language are attributed to a strong interaction between the semantics and phonological systems owing to the transparent orthography (alpha-syllable script or "aksharamala" in Kannada). Simultaneously, the reading deficits in the Kannada language result from deficits in these systems. This study highlighted the influence of non-reading abilities, such as semantics, phonology, and syntax (linguistic abilities) on reading abilities, highlighting the inherent interaction between linguistic modalities in reading proficiency specifically in the Kannada language. Research on language and reading rehabilitation in Dravidian language-speaking aphasics is in preliminary stages. Most investigations have predominantly centered on individual cases and have often lacked thorough pre- and post-rehabilitation assessments of linguistic and reading impairments using equivalent tests in the specific language. The study has effectively attempted to explore the linguistics and reading components in Kannada-speaking post-stroke survivors. The study serves to be preliminary in its attempt to understand the effect of linguistics on reading abilities in the Kannada language. Far more investigations are crucial with respect to bilingual contexts, errors in atypical reading patterns, and other contributing factors like rehabilitation, proficiency, and so on. 

Limitations

The observations drawn from the study should be approached with caution due to the limited sample size, and not considering the severity and type of aphasia manifested by the cohort in the study. A few hidden and uncontrollable variables interfering with the findings could be, language exposure, language usage post-stroke, limited language rehabilitation received in both languages, and other social limitations. Thus, further research must garner more comprehensive insights from larger samples encompassing various types of aphasia and severity of impairment.

## Conclusions

Alexia in post-stroke survivors is a most debilitating and prevalent condition, resulting in restricted social life participation. With the growing dependence on social media through messages texts, and emails, persons with aphasia feel the need to participate in social events through these modes. The study effectively reinforces the principles of primary system approaches. Initially developed to elucidate reading and acquired alexia, the primary systems approach characterized reading challenges as disturbances in cognitive elements that transcend mere reading proficiency. This current study has convincingly shown that performance on reading-related tasks reflects the functionality of central semantic, phonological, and syntactic processing elements. Given that literate adults routinely interact with both spoken and written language, a comprehensive assessment framework of language processing must encompass both modalities (linguistic and reading) for individuals with alexia.

## Appendices

Appendix A

Task-Specific Instructions and Details

The study protocol is broadly divided into investigating:

a) Non-orthographic linguistic tasks specific to Kannada and English language, which includes semantics, phonology, and syntax.

b) Oral reading tasks at single word level, specific to Kannada and English language, which includes real word reading, irregular word reading, and non-word reading.

c) Oral reading comprehension across single word level, sentence level, and paragraph level in both Kannada and English language.

1. *Semantic Tasks*

a) Picture association task: In this task, participants will be presented with four foils and a target picture, all in the form of printed picture flashcards. The participant will be expected to match the target picture with foil of four picture flashcards on three types of associations namely, distinctive feature association (camel as target word - dessert, water, pet, and forest); categorical association (apple-orange) and noun verb (bat-hit or scissor-cut) association.

b) Picture matching/identification task: Participants will be presented with four printed picture flashcards and they are instructed to match or identify the spoken word (target stimulus) to picture out of four foils. The items assessed in this task will comprise of common nouns and common verbs. For example; the target verbal stimuli will be "knife" and the participant has to identify out of four picture flashcards; "table," "knife," "pen," and "grapes."

c) Auditory synonym judgment task: Participants will be presented with pairs of words related in terms of synonyms through auditory mode by the researcher. They are instructed to indicate "yes" or "no" for the relatedness of pairs. For example; "look-see," "happy-excited," and "speak-tell."

d) Auditory comprehension task: Participants will be presented with polar questions through auditory mode and they will be expected to answer "yes" or "no" to the questions. For example; "sky is blue is color" (polar question) - "yes" (expected answer).

2. *Phonological Tasks*

a) Minimal pair judgment: Participants will be presented with minimal pair words verbally, and they will be instructed to judge if they sound the same or distinct. For example; "pat-bat," "mall-ball," etc.

b) Real word rhyme judgment: Participants will be presented with real rhyming words verbally, and they will be instructed to judge if they sound the same or not. For example; "mission-passion," "transform-inform" and so on.

c) Non-word rhyme judgment: Participants will be presented with rhyming non-words verbally, and they will be instructed to judge if they sound the same or not. For example; "Toption-Bension" and so on.

d) Parsing/blending sounds: Participants will be instructed to join the parts or sounds and produce the target words verbally in terms of phoneme isolation and syllable level. For example; isolation parsing: /p/ - /pat/, blending: /m/ + /mat/; syllables parsing: /pla/ - /plate/, blending: /pla/ + /ate/.

3. *Syntax Tasks*

a) Comprehension of plural forms and tense markers: Participants will be presented with pairs of words varying in their respective plural forms and tense forms. The participants are instructed to respond if the pair is in the correct form to the target word or not. For example: "table-tables," "child-children" (plural forms); "come-came," "fall-fell," "want-wanted" and so on.

b) Spoken sentence to picture matching: Participants will be presented with four picture flashcards and a sentence will be presented verbally. The participant is expected to match verbal sentences to respective picture forms. 

c) Sentence completion with locatives: Participants will be presented with a target picture, subsequently an incomplete sentence will be presented verbally. The participant is expected to fill in with appropriate locative form (in, on, over, under and so on) respective to the picture stimulus and the oral sentence. For example: "The apple is ………. (on) the table."

4. *Oral Reading Tasks*

Participants will be presented with a set of printed flashcards of single words (orthographic) and they are instructed to read aloud each word on presentation. All the words will be matched for length and complexity. For each item, they will be given 15 seconds time to read. The following words will be assessed in this task;

a) Real word reading (Kannada and English).  Example: /se:bu/ and "table."

b) Non-word reading (Kannada and English). Example: /hampara/ and "ganter."

c) Irregular word reading (only in English). Example: "yatch."

5. *Reading Comprehension (Silent) Tasks*

a) Single word level: All participants will be assessed on this task through various sub-tasks like

i. Written word-to-picture matching: Participants will be instructed to read the written word in the flash card and match it with the respective picture out of a choice of four pictures;

ii. Written synonym judgment: Participant will be presented with a target word in written form on a flashcard and instructed to match with the respective synonym to the word out of four printed word flashcards. Example: "happy-joy";

iii. Written word and semantic association: participants will be presented with target word in written (printed) form on the flashcard and instructed to match with the respective semantically related word out of four written (printed) words. Example: "Orange" target word - "apple, ball, water, tangle."

b) Sentence level: All participants will be assessed on this task through various sub-tasks like

i. Written sentence to picture matching: Participant will be instructed to read the written sentence in the printed card and match the respective picture out of a choice of four pictures;

ii. Written sentence judgment: Participants will be presented with correct and incorrect form of sentences in printed form and asked to judge for the correctness of the sentence on reading;

iii. Written sentence completion: Participants will be presented with incomplete sentence in printed form and they are instructed to fill in with appropriate form. "The sky is ………. in color."

iv. Written sentence conceptual matching: Participants will be presented with target sentence in printed form on one flashcard and will be instructed to match with other set of printed sentences which are conceptually related to the target sentence. For example: Target sentence - "When the sun rises" - "it lights up the whole world."

c) Paragraph level: All participants will be assessed on this task through various sub-tasks like

i. Paragraph-to-picture matching: Participant will be instructed to read the written paragraph (print form) in the flash card and match the respective picture out of a choice of four pictures

ii. Paragraph thematic matching: Participant will be instructed to read the written paragraph (print form) in the flash card and match the respective picture out of a choice of four pictures which are related to the theme of the printed paragraph.

iii. Paragraph inferential matching: Participant will be instructed to read the written paragraph (print form) in the flash card and match the respective picture out of a choice of four pictures with respect to the inference of the printed paragraph.
